# Service delivery reform for maternal and newborn health in Kakamega County, Kenya: study protocol for a prospective impact evaluation and implementation science study

**DOI:** 10.1186/s12889-022-13578-y

**Published:** 2022-09-12

**Authors:** Kevin Croke, Anna Gage, Isabel Fulcher, Kennedy Opondo, Jacinta Nzinga, Benjamin Tsofa, Sebastien Haneuse, Margaret Kruk

**Affiliations:** 1 Harvard T.H. Chan School of Public Health, Boston, MA USA; 2 Harvard Medical School, Boston, MA USA; 3 Harvard T.H. Chan School of Public Health, Kisumu, Kenya; 4 Kenya Medical Research Institute (KEMRI)/Wellcome Trust Research Programme, Nairobi, Kenya

**Keywords:** Maternal mortality, Neonatal mortality, Quality of care, Kenya

## Abstract

**Background:**

Maternal and neonatal mortality remain elevated in low and middle income countries, and progress is slower than needed to achieve the Sustainable Development Goals. Existing strategies appear to be insufficient. One proposed alternative strategy, Service Delivery Redesign for Maternal and Neonatal Health (SDR), centers on strengthening higher level health facilities to provide rapid, definitive care in case of delivery and post-natal complications, and then promoting delivery in these hospitals, rather than in primary care facilities. However to date, SDR has not been piloted or evaluated.

**Methods:**

We will use a prospective, non-randomized stepped-wedge design to evaluate the effectiveness and implementation of Service Delivery Redesign for Maternal and Neonatal Health in Kakamega County, Kenya.

**Discussion:**

This protocol describes a hybrid effectiveness/implementation evaluation study with an adaptive design. The impact evaluation (“effectiveness”) study focuses on maternal and newborn health outcomes, and will be accompanied by an implementation evaluation focused on program reach, adoption, and fidelity.

**Supplementary Information:**

The online version contains supplementary material available at 10.1186/s12889-022-13578-y.

## Background

Maternal mortality is 40 times higher and neonatal mortality is 9 times higher in low-income countries than in high-income countries [1], the Millenium Development Goal target of reducing maternal mortality by 75% by 2015 was not achieved [2], and the current rate of maternal mortality reduction (-2.9% per year from 2000–2017) will need to more than double to achieve the Sustainable Development Goal (SDG) target [1].

Progress against neonatal mortality, which now accounts for 46% of all under-5 deaths, is similarly off track, with more than 60 countries unlikely to achieve the SDG target of a neonatal mortality rate of 12 per 1,000 births or lower by 2030 [3]. Current global strategies to reduce maternal and neonatal mortality in developing countries largely focus on extending the reach of decentralized, clinic-based antenatal and delivery care to populations that still lack access to this care [4]. A key element of this strategy has been to increase access to antenatal care and to increase the percentage of women who deliver in a health facility or with care from a skilled birth attendant. These indicators have improved rapidly in many countries. However, these increases have not resulted in commensurate reductions in maternal and neonatal mortality [1]. This suggests that access to basic care is not enough. Improving access to *high quality, definitive* maternal and newborn care is critical for reducing maternal mortality, neonatal mortality and stillbirths, reducing maternal and neonatal morbidity, and achieving SDG 3 targets.

One reason why quality remains inadequate for intrapartum care is that many facility deliveries take place in poorly equipped primary care clinics that do not consistently diagnose high-risk pregnancies, are not equipped to manage intrapartum complications, and do not have systems in place to consistently refer severe cases to hospitals in time [5]. Across a range of low and middle-income countries, quality of delivery care is better in higher-level facilities, while lower level facilities and low case volumes are often associated with low quality [6]. Recent evidence shows that in the absence of advanced care (such as well-equipped and staffed surgical theaters and newborn units), point-of-care quality interventions at primary care level are unlikely to save lives [7]. Other recent observational evidence shows limited effect on maternal or newborn mortality from low-level facility delivery in low income settings [8, 9].

The linked crises of maternal and early neonatal mortality, and the inadequacy of current strategies, highlight the need to accelerate progress by developing and testing new approaches to delivery and newborn care. The recent Lancet Global Health Commission on High Quality Health Systems [10] argued for a structural health system reform in high mortality settings with adequate geographic access to hospital care: shifting the default location for deliveries from primary care clinics to newly strengthened and expanded “delivery hub” hospitals equipped to provide caesarean sections, blood transfusions, focused care for sick newborns, and other advanced services. The proposed policy, known as “Service Delivery Redesign for Maternal and Newborn Health,” (SDR) envisions that prior to any shift in recommended delivery location, health system leaders would improve the quality of care available in delivery hubs, ensure adequate transportation options, strengthen networks of care between higher and lower-level facilities, and engage communities around the policy change. However, while there is increasing interest in the concept of SDR for maternal and newborn health, there is little rigorous evidence about the effectiveness of a shift to this proposed strategy in diverse low-income settings with scarce health system resources, a legacy of primary care delivery, and potential access challenges [11].

Kakamega County in western Kenya has decided to pilot a version of this policy: the county has begun to bolster, expand and equip “delivery hub” hospitals while improving emergency transportation access and granting facilities increasing scope to retain and manage locally-generated financial resources. After this period of system improvement focused on delivery care, the policy will be changed such that women will be strongly encouraged to deliver at nearby “delivery hub'' hospitals, rather than in primary care clinics (known as level 2 or level 3 health centers in Kenya). These interventions and policies represent a locally-adapted version of the SDR model. This protocol discusses the research design for a prospective study of the impact of SDR. The study comprises both an impact evaluation (“effectiveness”) component focused on maternal and newborn health outcomes, accompanied by an implementation evaluation focused on program reach, adoption, and fidelity.

## Methods

### Setting

Kakamega County is located in Kenya's Western Region, approximately 50 km from the Lake Victoria port of Kisumu. With a population of approximately 1.9 million, the county's maternal mortality rate was 316 per 100,000 live births and the neonatal mortality rate was 19 per 1,000 live births in 2014, when the most recent data is available [12]. There were approximately 70,000 births in the county in 2018; 35% of which occurred at home, 28% in primary care facilities (dispensaries (Level 2) and health centers (Level 3)) and 37% in hospitals (Level 4 and 5 facilities) [13]. There are 205 government health facilities in the county, 10 of which are Level 4 hospitals. Maternal and newborn care is free in most public health facilities throughout Kenya via the “Linda Mama'' program; additionally, Kakamega County introduced a conditional cash transfer program (“OparanyaCare'') for antenatal, delivery, postnatal care and immunizations to poor and vulnerable women.

### The intervention

SDR for maternal and newborn health is a multi-component health system reform. In Kakamega, SDR is being implemented by the County Department of Health, with support from the non-governmental organization (NGO) Jacaranda Health. Financing is from the County government and is supplemented by the Bill and Melinda Gates Foundation.

An initial feasibility assessment study examined the existing capacity, facility accessibility, political will and potential benefits of SDR in Kakamega County [13]. Based on this assessment, the County identified several components for a health system reform. First, it selected ten hospitals to serve as delivery hubs across the county. These were all public facilities; typically sub-county hospitals (level 4 hospitals in the Kenyan system). Before the intervention, the delivery hubs all conducted 1,000 or more deliveries per year, but not all had comprehensive emergency capabilities.

**Fig. 1:**
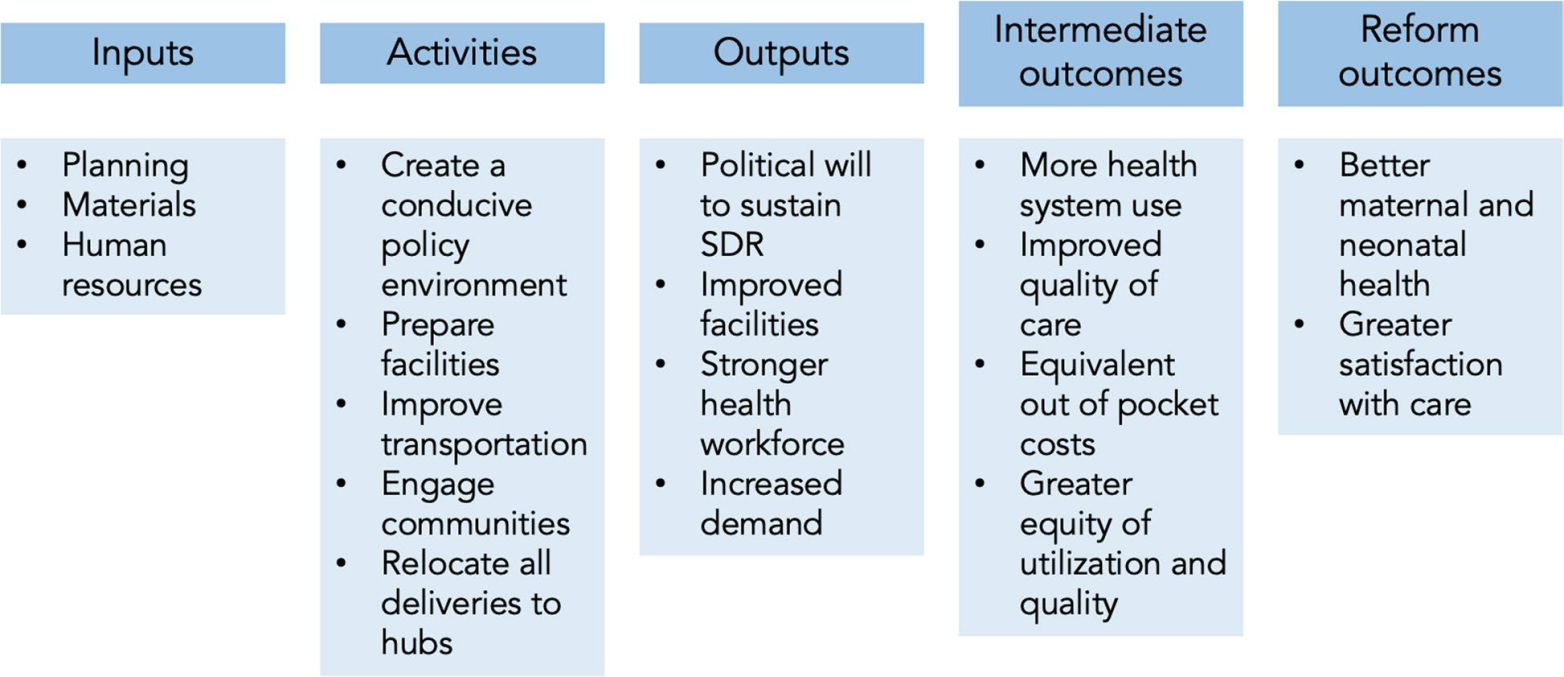
Service Delivery Redesign Theory of Change

The first component of SDR is a preparation phase, in which delivery hubs are strengthened (Fig. 1) and prepared for the higher patient volumes that will result from the SDR policy change of encouraging delivery at delivery hubs. This includes deployment of additional staffing, as well as improvements to infrastructure and equipment. For example, designated delivery hub hospitals in Kakamega are scheduled to be staffed with additional nurses, doctors, and specialists: In addition to their pre-existing complement of medical officers, the current plan is for each delivery hub in Kakamega to have a surgeon, a pediatrician, an obstetrician-gynecologist, and a specialist medical officer posted to the facility to bolster delivery care.

On the infrastructure side, each delivery hub will have operating theaters built or improved, maternity wards expanded, and newborn units built or expanded. Facility capacity to deliver blood transfusions will also be augmented.

Delivery hubs are further from most women's homes than local clinics; traveling greater distances to deliver can be difficult, especially at night. In Kakamega's SDR reform, this issue will be addressed with the participation of the NGO Rescue.co, who contracts with private transport providers to provide emergency transport for pregnant women.

When delivery hubs are upgraded to a level that will enable higher patient volumes and improved quality of care, and complementary interventions such as emergency transport and facility financing reforms are in place, the government of Kakamega plans to communicate to all health workers, and all health system users, that policy regarding place of delivery has changed, and women are now expected to deliver at designated delivery hub hospitals. To this end, the last component of the intervention is this public communication element, which will be undertaken through media campaigns and public outreach meetings, as well as direct communication from health workers to antenatal care (ANC) clients.

### Research questions

#### Impact evaluation component

The main research question of the impact evaluation (effectiveness) component of this study is the impact of SDR in Kakamega County on maternal and newborn health, measured using a composite indicator of maternal and newborn health. The secondary research question examines the impact of SDR on patient satisfaction and perceived quality of care, maternal complications, out-of-pocket (OOP) expenditures, and utilization of care.

#### Implementation component

The main research objective for the implementation component of the evaluation will be to measure the implementation fidelity and reach, identify key obstacles to successful implementation as well as facilitating factors, and measure possible unintended consequences of SDR policy on health system functioning.

### Study design

This protocol describes two study designs for the impact evaluation component of the SDR evaluation: the first is a multi-phase stepped wedge design, for the planned situation in which SDR is progressively scaled up across all of Kakamega County’s sub-counties in three phases. However, due to the multi-year, complex nature of the SDR intervention, we also pre-specify a smaller scale, nested “phase 1'' study design which will be carried out for two reasons. First, a study based on phase 1 will be conducted to track a set of implementation indicators in order to assess policy implementation. As a pragmatic, real world health system evaluation, findings about implementation and take-up from this first phase can be used strengthen phase 2 and phase 3 implementation. Second, in the event that only the first phase of the reform is fully implemented, the full set of outcomes will be analyzed after phase 1 using a difference-in-difference design.

In the sections below we first discuss the full SDR reform impact evaluation (“effectiveness evaluation”), and then discuss the phase 1 sub-study. Then, we discuss the implementation science component of the study.

#### Full policy evaluation

**Fig. 2:**
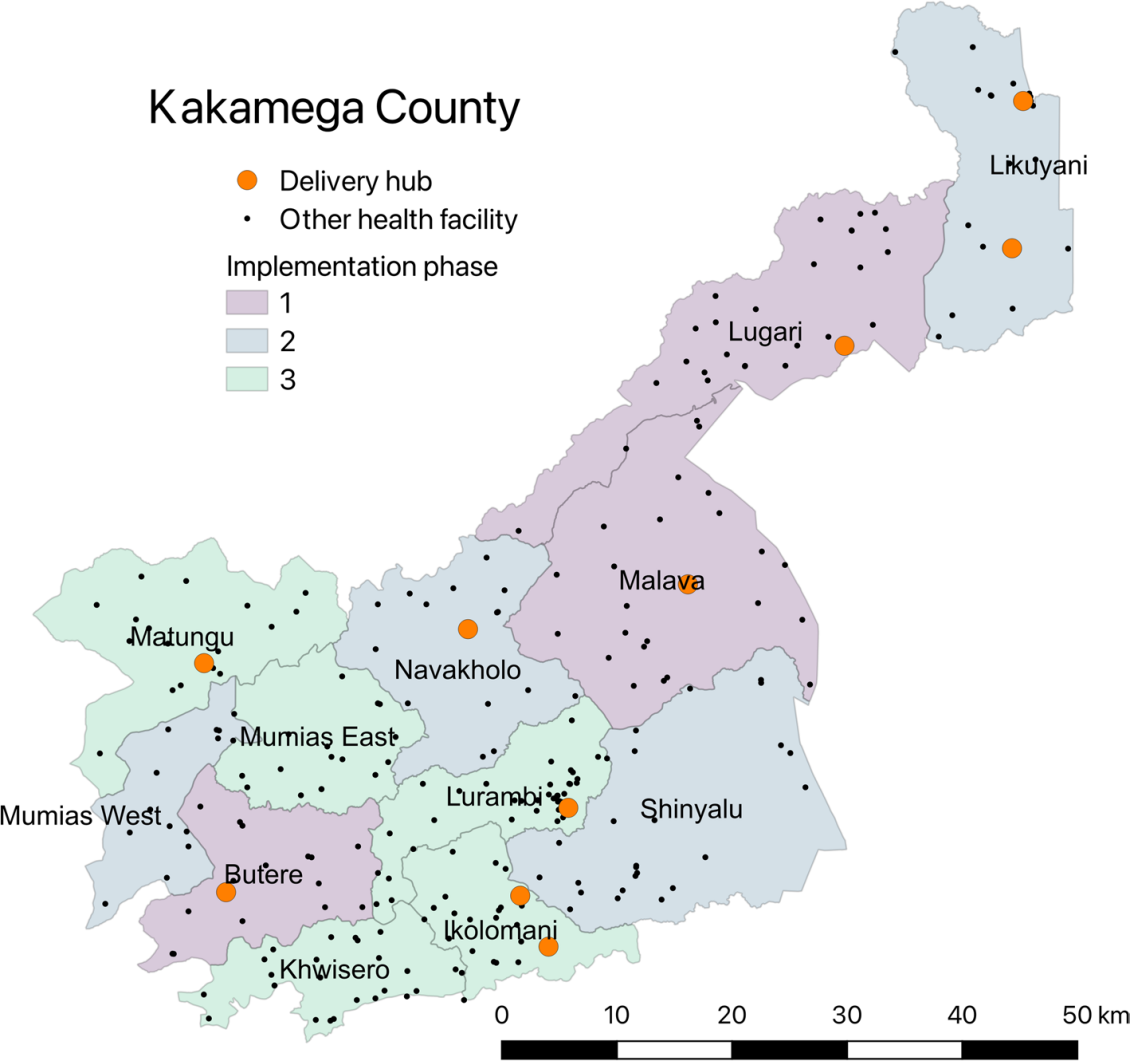
Phases of Service Delivery Redesign implementation by sub-county in Kakamega County (Source: authors)

This study will use a prospective, observational stepped wedge design. Kakamega County is planning to implement a phased rollout of SDR by sub-county, in three phases. Kakamega has 12 sub-counties: the reform will be implemented first (starting in 2022) in a group of 3 sub-counties. After a period of implementation in these “phase 1'' sub-counties, a second set of 4 sub-counties will implement the reform. After another period of implementation in these phase 2 sub-counties, a final set of 5 sub-counties will implement the reform (Fig. 2). This phased approach parallels the structure of a stepped wedge trial in that delivery and post-delivery outcomes for mothers and newborns can be compared within each group of sub-counties that implement SDR in the same implementation phase before and after the roll-out, as well as across counties in the same phase of rollout. Analytically, the analysis is also similar to that of a stepped wedge cluster randomized trial; the difference is that the order in which the clusters are exposed to treatment was decided not via randomization, but was decided by Kakamega County policymakers.

While noting the planned three phase design, we also have included an adaptive element to the evaluation study, due to the complex nature of the intervention: SDR is a multi-element, health systems intervention, requiring significant investment in infrastructure, human resources, and equipment. Furthermore, much of the investment is being executed using the county's public budget (in addition to contributions from the Gates Foundation). Given the public component of SDR spending and implementation through public sector institutions, the research team cannot fully anticipate the timing of the various elements of the stepped wedge design. Therefore in this trial we specify a secondary, interim design which leverages the first phase of SDR, described further below.

#### First phase interim evaluation

Embedded within the planned stepped wedge design is a difference-in-difference interim study design with the intervention occurring in phase 1 sub-counties (equivalent to the first “step'' or wedge in the planned stepped wedge sequence). This multi-stage design will enable the research team to evaluate interim results of the reform after phase 1. The design will also enable a full evaluation to be conducted even in the event that only one phase of the policy is implemented. In the event that the reform is only implemented in phase 1 subcounties, the research team will conduct an interim study after phase 1 implementation is completed, while also conducting a difference-in-difference study comparing outcomes in phase 1 subcounties to phases 2 and 3 subcounties over the full two year evaluation period.

### Participants

**Fig. 3:**
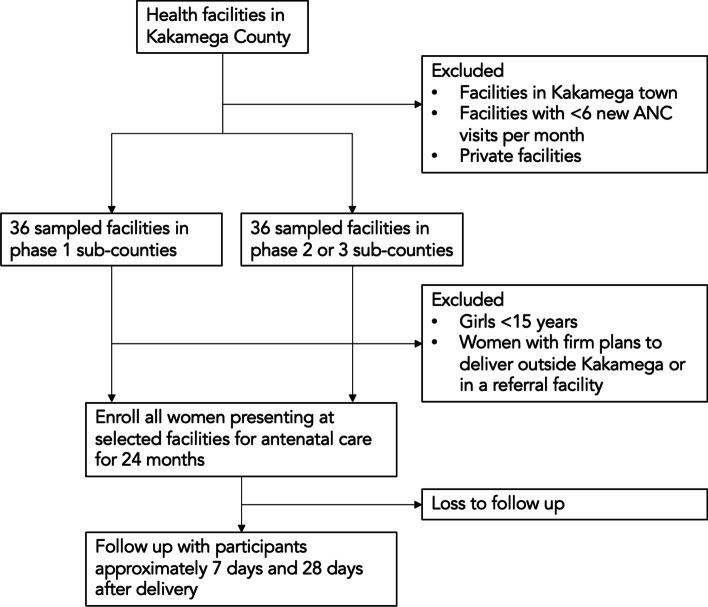
Participant flow diagram

The research team has begun to enroll study participants from a sample of 72 facilities which provide ANC across all 12 sub-counties of Kakamega (enrollment started in February 2022) (Fig. 3). The selected facilities were stratified by sub-county and randomly selected proportional to volume of antenatal care visits, excluding facilities located within Kakamega Town (defined as three most densely populated wards in the vicinity of Kakamega Town). Sampled facilities were divided evenly between those in the three Phase 1 intervention sub-counties (36 facilities) and the other nine sub-counties (36 facilities). All private facilities were excluded, as the main SDR intervention components are not planned to take place in these facilities. Finally, facilities with the extremely low ANC volumes (< 6 new ANC clients per month) were excluded from the sample. These represent the lowest quartile of facilities by ANC volume in the county.

In each sampled facility, survey firm staff working with the research team seeks to enroll all pregnant women who present for antenatal care over a two year period (6 months of baseline and 18 months of implementation).^1^ Based on administrative data from 2021, we estimate that this sampling strategy will yield a sample of 59,753 mothers across the 72 facilities over two years. Approximately 98 percent of pregnant women in Kakamega County receive at least one ANC visit (National Bureau of Statistics et al. 2015). The approximate annual number of births in Kakamega County is 61,442, so over the two years of data collection approximately half of all pregnant women will be enrolled in the study. Any woman who reports that she intends to give birth outside at Kakamega County at the time of enrollment will be excluded. In addition, women who state upon enrollment that they have already made firm plans to deliver at a referral facility due to complications or previous caesarean sections will be excluded. Finally, all pregnant women below 15 years of age will also be excluded.

### Data

The data collection strategy is informed by the SDR theory of change (Fig. 1), which has multiple pathways. According to the reform logic, in sub-countries where SDR has been implemented, pregnant women will change their care-seeking behavior by shifting their place of delivery from home or primary care clinics to delivery hub hospitals. The extent of this shift in care-seeking behavior will be measured by the main survey of enrolled pregnant women, hereafter referred to as the *pregnancy registry*. A second component of the theory of change is that delivery hubs, having been upgraded with better equipment, infrastructure, and staff, will deliver high quality intrapartum, postpartum, and newborn care. For the subset of women and newborns with life-threatening conditions (obstructed labor, sepsis, postpartum hemorrhage, low birthweight, birth asphyxia), timely and appropriate treatment will save lives, resulting in less maternal and newborn mortality and morbidity. We will measure both elements of this theory of change. Place of delivery and ultimate health outcomes will be measured through the pregnancy registry. Quality of care provided will be measured through facility and provider surveys, direct observations of deliveries, and women’s reports of quality from a subset of the main survey.

#### Pregnancy registry

The main data collection tool to measure the primary outcomes will be the pregnancy registry survey, which includes both enrollment at ANC and post-delivery follow-up surveys. All eligible, consenting pregnant women presenting for ANC in the 72 participating facilities (except the excluded categories mentioned above) will be enrolled.^2^ At this visit, basic demographic information, anticipated delivery date, preferred and previous delivery locations, self-reported and formally diagnosed antenatal complications, and detailed contact and tracking information will be collected. Participants will be contacted by phone 7 days after their anticipated delivery date and again 28 days after their expected delivery date. In these interviews, details regarding maternal health and child survival and mother’s delivery experience will be collected, as well as mothers’ receipt of post-natal care, satisfaction with delivery care, and OOP costs for delivery including transportation costs. In-person follow up visits will be conducted (using tracking information collected at enrollment) if respondents cannot be reached by phone.

#### Longitudinal survey

A subset of 2,000 respondents from the pregnancy registry will be invited to take part in a more detailed follow up with multiple interviews over the course of their pregnancy and immediate postpartum period. Interviews for this survey (known as the *longitudinal survey*) will take place in a random subset (40 out of 72) of sampled facilities. Approximately 3% of all enrolled women will be randomly sampled within the pregnancy registry at enrollment for participation in the longitudinal survey. This survey will be conducted, in person, at the antenatal care facility at enrollment; over the phone during the month 8 of pregnancy, 7 days after her anticipated delivery date, and 28 days after delivery; and again in person at the participant’s home 2 months after delivery. These surveys will allow deeper investigation of topics including: previous pregnancies, quality of antenatal, delivery and post-natal care, delivery location intention at various points in pregnancy and reasons for same, travel modality and time to delivery hubs, socioeconomic status, awareness of SDR, and opinions about the health system. Basic anthropometrics and biomarkers (height/weight, blood glucose, hemoglobin and blood pressure) and will also be collected at enrollment and at the final follow up. For newborns, anthropometrics will be captured at the final follow up visit.

#### Delivery hub facility surveys, provider assessments, and delivery observations

Facility audits and provider assessments will be conducted in planned delivery hubs and all other level 4 and level 5 hospitals (*n* = 14) four times over the study period: once six months before the start of phase 1, once at the start of each of the three waves of SDR implementation. These surveys will measure capacity for deliveries, structural quality, and process quality of care of each delivery hub before and after re-design is implemented. For example, the survey will measure the number of maternity beds, the presence of a newborn unit, the number and functionality of operating theaters, presence of staff (including all medical doctors by specialty, as well as clinical officers, nurse-midwives, nurses, and other cadres), the availability of 24 h care, the presence of both basic and comprehensive emergency obstetric signal functions including procedures, equipment, and medicines. These surveys will also measure provider knowledge and attitudes both before and after the redesign, with particular focus on the management of common obstetric complications, and will also assess working conditions, motivation, and awareness of SDR implementation. All providers who are working in the maternity or newborn care units the day of the facility assessment will be invited to participate in the survey.

Enumerators who are trained in doing deliveries (i.e. nurses or midwives) will conduct continuous observations of deliveries for one week in each delivery hub (*n* = 10) at four times over the study. Observations will be used to assess the technical and interpersonal quality of care from the time women are admitted to the labor ward until approximately 6 h after her delivery or her discharge from the facility. Observations will be conducted 6 months before the start of phase 1, and once at the start of each of the three waves of SDR implementation. Given current volumes in delivery hubs from administrative data, an estimated 1,500 deliveries will be observed over the course of the study.

#### Primary care facility survey and provider assessment

Facility and provider surveys will also be conducted in a random sample of 40 primary care (i.e. non-delivery hub) facilities will take place twice: during the baseline period and at the end of phase 1. The purpose will be to examine the capacity and readiness of antenatal care providers and facilities, their skills, job satisfaction, and the resources, drugs, and equipment that they have to do their work, and how this changes over the initial period of SDR implementation. All providers that provide antenatal, postnatal, or maternity care who are working the day of the facility assessment will be invited to participate.

### Outcomes

#### Impact evaluation: primary outcomes

The primary outcome of this study is a composite measure of maternal and neonatal mortality. This composite was selected, following similar trials (e.g. Semrau et al. 2017) because SDR is hypothesized to improve both maternal and neonatal outcomes, and because the individual components of this measure would likely not occur frequently enough to allow us to reliably detect changes within the population of Kakamega County. Compared to the composite used in similar trials [7], we omit the “maternal near miss” element from the composite outcomes, due to the difficulty is accurately assessing near miss based on retrospective self report [14]. We estimate a baseline prevalence of the composite indicator of 4% in Kakamega County.

This composite measure reflects severe maternal, fetal, and neonatal adverse events that occur around the time of childbirth relative to the total number of birth events. For this study, a birth event is defined as the period from the time of maternal admission for childbirth (or the onset of labor, for home deliveries) until the completion of 28 days after birth. A birth event considers the mother and baby to be a single dyad given that their health outcomes are many times interdependent. For this reason, multiple gestations will also be considered to be a single birth event. A birth event may result in a stillbirth and/or a live birth.

Specific outcomes comprising the composite measure are:

A. Maternal death at 28 days: defined as death of a woman at any time from onset of labor, through delivery, until the period of 28 days following delivery.
B. Intrapartum stillbirth: defined as death during labor >  = 28 weeks gestation, as evidence by lack of maceration and by a reporting of fetal movements in the two days before labor.
C. Neonatal mortality: defined as a newborn death that occurs within the first 28 days of life.

The composite outcome will be assessed through the two follow up phone surveys from the pregnancy registry, and in person surveys when respondents cannot be reached by phone.

#### Impact evaluation: secondary outcomes

Secondary outcomes of the study include patients’ satisfaction with care received and confidence in the health system, the location of care (health center versus hospital), OOP expenditures, quality of delivery care, travel time, self-reported maternal complications, and equity on all of these dimensions between women.

Women’s satisfaction with the care that they receive will be measured in the pregnancy registry, and with more detail, in the longitudinal survey. Total OOP expenditures will be measured using seven questions capturing expenditures on transportation, overnight lodging, medical fees, registration, drugs and supplies, medical tests, food, and any other costs. These will be summed to create an OOP expenditures indicator. Quality of care will be measured using both women’s perceived quality as well as through observations of care in delivery hubs. Perceived quality ratings will include women’s ratings on technical quality and respectful care quality for delivery as well as for ANC and postnatal care (PNC). Using the observations of delivery care, technical quality of routine care will be assessed through a 20-item index capturing the processes of intrapartum and immediate postpartum care [15]. Respectful care will be measured using a 10 item index measuring provider actions, such as whether or not a provider explains procedures before proceeding [16]. Care utilization will be measured with both the facility utilization rate, the delivery hub utilization rate, as well as ANC and PNC utilization, using the pregnancy registry. Additional data from the Kenyan Health Information System (KHIS) will be used to compare delivery hub utilization rates between enrolled and non-enrolled pregnant women during the study period. Maternal complications will be defined through the self report of one of the following: fits (in absence of history of epilepsy), loss of consciousness for > 1 h, high fever with chills and foul-smelling discharge, blood transfusion, hysterectomy, intensive care unit admission, mechanical ventilation, or hospital stay of > 7 days. Gaps in access and outcomes by education, proximity to delivery hubs, and household socio-economic status will be compared for the satisfaction, utilization, and cost outcomes described above in implementation sub-counties versus control sub-counties.

#### Implementation evaluation: direct and indirect outcomes

For the implementation science component of the study we will measure coverage, adoption, and fidelity of SDR implementation using survey, administrative, and program data on activities and outputs. We will track indicators on program reach (e.g. percentage of primary care clinics where women are routinely advised to deliver at delivery hubs; percentage of women who report being advise to deliver in delivery hubs); adoption (e.g. percentage of women delivering at delivery hubs; percentage of delivering at health centers; labor and delivery quality of care; ANC and PNC utilization and quality; C-section rate); and intervention fidelity (maternity ward and newborn unit expansions in delivery hub hospitals; specialist physician and nurse staffing levels; maternity and newborn staff knowledge levels; availability of critical inputs such as blood for transfusions).

Given the size and scope of the policy reform, it is likely that SDR will affect the health system in indirect ways, beyond the hypothesized effects on maternal and newborn health services. Complex system-level reforms can have both positive and negative broader systemic effects. Therefore we will measure potential system-level effects of SDR as secondary implementation outcomes (Table 1).

**Table 1:** Implementation evaluation indicators

Direct effects: Implementation evaluation		
Reach	% ANC clinics where women are routinely advised to deliver at delivery hubs	Primary care facility survey
	Percentage of women who report being advised to deliver in delivery hubs	Longitudinal survey
Adoption	% women delivering at delivery hubs	Pregnancy registry; KHIS
	% women delivering at primary care facilities	Pregnancy registry; KHIS
	Delivery quality of care	Delivery observation checklist
	ANC and PNC utilization	Pregnancy registry; KHIS
	Caesarean section rate	Pregnancy registry; KHIS
Fidelity	Maternity ward and newborn unit capacity in delivery hub hospitals	Delivery hub survey
	Maternity and newborn staff knowledge levels	Delivery hub and primary care provider assessments
	Availability of critical inputs (e.g. blood for transfusions)	Delivery hub facility survey
	Specialist physician and nurse staffing levels	Delivery hub facility survey
Indirect effects: Implementation evaluation		
Indirect effects	Utilization for non-MNCH services (e.g. HIV, TB) from hospital congestion	KHIS
	Utilization of private sector for deliveries	KHIS; Pregnancy registry
	Continuity of ANC, delivery, and post-natal care	Pregnancy registry
	Surgical volumes for non-MNCH conditions	KHIS
	Health worker morale and retention	Delivery hub and primary care provider assessments
	Delivery hub congestion/overcapacity	KHIS

*MNCH:* Maternal, Newborn and child health, HIV: Human Immunodeficiency virus, *TB:* Tuberculosis

### Power calculations

For the impact evaluation evaluation component, we performed simulation-based power calculations for the composite outcome in the stepped wedge evaluation design to determine the number of facilities to sample. We excluded private facilities, facilities in Kakamega town, and facilities with less than or equal to 6 first antenatal care visits per month. There were 94 facilities in control sub-counties and 59 in treated sub-counties.

We assumed that the baseline composite outcome was 0.0406. Given that direct observation of the primary composite health outcome was not available at baseline, we estimated it using three strategies and selected the most conservative estimate (40.6 events per 1000 births) based on a prospective cohort study of maternal death, stillbirth and neonatal deaths with a site in Western Region of Kenya encompassing 31,118 births [17]. We then generated outcome data across Kakamega’s health facilities by:

1 $$logit\left(P\left({Y}_{ik }= 1 \right| trt, {\gamma }_{0k }\right)= {\beta }_{0}+ {\beta }_{1}{trt}_{ik}+ {\gamma }_{0k }$$

where *k* indicates sub-county, *Y* is composite outcome occurrence for delivery *i* in for a women living in sub-county *k* and *trt* takes value 1 or 0 depending on if the cluster is receiving the intervention during that phase. We also include a random intercept ${\gamma }_{0k}$ for each sub-county drawn from a normal distribution with standard deviation 0.20, which was based on an assessment of expected variation in composite outcome rates between sub-counties.

To determine the appropriate number of facilities to sample, we calculated the power varying the number of facilities sampled from the treated and control sub-counties. We generated 1,000 simulated datasets followed the planned sampling design by first stratifying by sub-county and then sampling facilities with probability proportional to the number of antenatal care visits in 2021. Over the full study (following the stepped wedge design in which all sub-counties ultimately receive the SDR reform), we determined that we would have 89% power to detect a 12.5% reduction in composite outcome (a drop from 0.0406 at baseline to 0.0355) for 72 facilities, roughly corresponding to 60,000 births. To optimize power in the difference-in-difference Phase 1 study, we determined that equal allocation of 36 facilities in Phase 1 sub-counties and 36 facilities in Phase 2 and 3 sub-counties (18 facilities in Phase 2 and 18 facilities in Phase 3 sub-counties) would result in 86% power to detect a 30% reduction in composite outcome (with 72% power to detect a 25% reduction). For our secondary outcome of satisfaction, we determined that 2,000 women from the longitudinal study sample would result in > 80% power to detect a 0.15-standard deviation increase for both evaluation designs.

### Analysis

#### Full SDR evaluation, primary analysis

The primary analysis will assess the impact of SDR on the composite outcome comprised of maternal mortality, neonatal mortality, and stillbirth. We will perform both an intention to treat and per protocol analysis.

Intention to treat analysis: We will fit the model in Eq. (1), as well as analogous linear models. A participant’s treatment status will be determined based on her sub-county of residence. If she lives outside of Kakamega County, it will be determined by the location of the facility where she is enrolled. Since the order in which subcounties were selected for SDR was not random, but rather was a decisions taken by Kakamega County policymakers, it is possible that respondents will differ on observable characteristics which may be correlated with the study’s outcomes of interest. We will account for sources of confounding by controlling for mother's education, age, parity, transport time to nearest delivery hub, season of pregnancy, and perceived pregnancy risk in our regression model. Self-reported pregnancy risk is captured by the following question “How concerned are you about complications during this pregnancy?” with a response scale of 1 (no concern) to 5 (very concerned) and will be coded as a binary variable indicating high risk (choice of >  = 4). Distance will be measured by approximate travel time from a woman's village to the delivery hub. Additional confounders from external data sources such as rainfall or temperature data may be incorporated in the model, depending on availability.

Per protocol analysis: We will compute the proportion of women living outside of intervention areas that are delivering at another sub-county's updated delivery hub. If this happens often (> 5% of deliveries in non-intervention facilities), we will also estimate the effect of delivery in an updated delivery hub on the composite outcome (instead of the effect of living in a treated sub-county). In addition to the confounders listed for the intention-to-treat analysis, we will adjust for whether the woman comes from an intervention area.

Account for missing outcomes data: We expect there to be loss-to-follow-up after delivery resulting in missing values for the composite outcomes. The survey team will attempt multiple phone-based and in-person tracking options before coding an outcome as missing. We will use multiple imputation to impute missing outcome values.

#### Secondary analyses

Secondary outcomes include satisfaction with care, respectful care index, out-of-pocket expenditures, location of delivery, travel time, maternal complications, and equity. These outcomes will be analyzed as above, with the exception the respectful care index and travel time outcomes, which will be restricted to the random sample of women in the longitudinal cohort. We will also analyze equity for selected outcomes (e.g. quality of care, OOP cost). In this analysis we will include an interaction term between delivering at an updated delivery hub and the equity markers to assess baseline disparities in outcomes and test if these disparities changed during the intervention.

Phase 1 interim analysis: After six months of data has been collected following the implementation of improvements in Phase 1, we will conduct a study on interim outcomes to understand implementation of the reform and how it has affected women's decision-making about delivery care. We will examine the effect of policy exposure on the same outcomes as in the full evaluation. The main analytical difference is that this analysis will be conducted using a difference-in-difference design rather than a stepped-wedge design as we will only observe the intervention among women living in Phase 1 sub-counties. Otherwise, the analytical approach will be the same for the Phase 1 study as for the full SDR evaluation.

Data collection will be monitored in an ongoing basis using the interim analysis described in Additional file 1: Appendix 1.

## Discussion

This study protocol describes a prospective impact evaluation and implementation science study to understand a novel policy reform to improve maternal and newborn health in Kakamega County, Kenya. The intent of the SDR reform is to shift all facility deliveries into ten equipped and capable delivery hubs that are equipped to provide definitive care in case of maternal or newborn complications. The study will aim to evaluate the impact of this reform on maternal and newborn health outcomes and a range of secondary outcomes on utilization, quality, cost, equity, and satisfaction, as well as understand the implementation of the reform. This research will create a comprehensive overview of the impact of SDR as well as the strengths and challenges faced in implementation.

This research will make a significant and novel contribution to the evidence base on health system improvements for maternal and newborn health. To our knowledge, this is the first study to prospectively study a reform that purposefully shifts deliveries into hospitals with the goal of improving health outcomes in a low or middle-income country. If SDR is successful in reducing maternal and newborn mortality and stillbirths in Kakamega County, the reform could be a model for other regions in Kenya and beyond. The findings will directly benefit mothers and newborns, provide guidance to County health executives in Kakamega County on maternal and newborn health policy, and inform global policymakers considering similar reforms.

### Strengths and limitations

This study has a number of strengths, including its prospective stepped-wedge design, large sample, and detailed measurement of the pathways by which the health outcomes may be affected. However, there are several limitations to consider. First, the reform is not randomized; the order of phased implementation was determined by the Kakamega County Department of Health team to maximize synergy with planned facility upgrades and other interventions. Second, implementation in the second and third phases of sub-counties is not assured and depends on additional financing and County commitment following Kenya’s 2022 elections. We account for this with the secondary difference-in-differences study design nested within the larger study. Third, as the sub-county is the natural administrative level for the intervention, there are a limited number of study clusters. Finally, the reliance on self-reported health outcomes may lead to measurement error in event rates. We plan to validate these self-reported health outcomes with facility records for a subset of women who deliver in a health facility to address this concern.

## Supplementary Information

**Additional file 1:** **Appendix.** Interim analyses.

## Data Availability

Data used in the power calcuations were provided by the Kenyan Ministry of Health. The data are available from the authors upon reasonable request and with permission of the Kenyan Ministry of Health.

## Abbreviations

ANC: Antenatal care
KHIS: Kenya Health Information System
NGO: Non-governmental organization
OOP: Out of pocket
PNC: Postnatal care
SDG: Sustainable Development Goals
SDR: Service Delivery Redesign

## Footnotes

1 The length of the baseline period may vary based, as it will depends on speed of phase 1 implementation.

2 During the baseline period, pregnant women will be enrolled on any ANC visit, provided that they have not been previously enrolled. Once the study has been running for the baseline period of 6 months or more, the majority of remaining unenrolled women attending study ANC clinics will be on their first ANC visit.
