# Supplementary material for: Service delivery reform for maternal and newborn health in Kakamega County, Kenya: study protocol for a prospective impact evaluation and implementation science study
Source: BMC Public Health. 2022 Sep 12;22:1727. doi: 10.1186/s12889-022-13578-y (PMC9465958; doi:10.1186/s12889-022-13578-y)
Supplement: Supplementary file 1 — Additional file 1: Appendix. Interim analyses. [file 12889_2022_13578_MOESM1_ESM.docx]

**Appendix**

*Interim analyses*

**Implementation monitoring:** After each 6-month phase is complete (including baseline), we will ensure that patient enrollment and follow-up is proceeding as expected via the following analyses:

- For each facility, report the proportion of women that: (1) declined participation, (2) lived outside of Kakamega county, (3) did not plan to deliver in a Kakamega facility.
- For each facility, compute the expected monthly number of antenatal care visits (using KHIS monthly data from prior years). Compare expected to the observed monthly number of ANC visits during each phase.
- Report the proportion of women that were unable to be reached. Compare demographic/clinical characteristics between women who are loss-to-follow-up to those who are reached.
- Summarize the distribution of: (1) the difference between estimated and actual date of delivery (2) the number of days after (or before) delivery that we contact women.
- In each sub-county, report the proportion of women delivering at their delivery hub, another county's delivery hub, lower tier facility, or at home.
